# AFTA-Net: Axial Fusion and Triaxial Factorised Attention Network for Nowcasting of Severe Convective Weather

**DOI:** 10.3390/s26051409

**Published:** 2026-02-24

**Authors:** Huantong Geng, Delong Fang, Xiaoran Zhuang, Liangchao Geng, Xinxin Zeng

**Affiliations:** 1 School of Computer Science, Nanjing University of Information Science and Technology, Nanjing 210044, China; htgeng@nuist.edu.cn (H.G.); 202412200736@nuist.edu.cn (X.Z.); 2 Jiangsu Meteorological Observatory, Nanjing 210008, China; zxrxz3212009@163.com; 3 School of Atmospheric Science, Nanjing University of Information Science and Technology, Nanjing 210044, China; genglc_nuist@163.com

**Keywords:** radar echo extrapolation, deep learning, spatiotemporal decoupling, feature recalibration, severe weather nowcasting, attention mechanism

## Abstract

Radar echo extrapolation is a core technique for 0–2 h nowcasting, yet existing deep learning models often struggle with non-linear atmospheric motion and intensity attenuation due to insufficient feature decoupling. To address these limitations, this paper proposes AFTA-Net, a novel encoder–decoder architecture. The model introduces an Axial Fusion Block (AFB) that employs a parallel decomposition strategy to explicitly separate temporal evolution from spatial morphology, preserving structural integrity while capturing motion trends. Furthermore, a Tri-Axis Factorized Attention (TAFA) mechanism is designed to sequentially recalibrate feature representations across Time, Channel, and Spatial dimensions, thereby enhancing sensitivity to high-frequency convective signals and suppressing background noise. Extensive experiments on the Jiangsu radar dataset demonstrate that AFTA-Net significantly outperforms representative baselines. Notably, at the critical 30 dBZ threshold for severe weather, the model achieves a CSI of 0.2506 and an HSS of 0.3430.

## 1. Introduction

Climate change has intensified the frequency and severity of extreme weather events globally, making accurate short-term weather forecasting, or nowcasting, increasingly critical for disaster mitigation and public safety [1,2]. Severe convective weather systems, such as squall lines and supercells, are characterized by rapid evolution and non-linear dynamics, posing significant challenges for prediction. Doppler Weather Radar, as a primary remote sensing instrument, provides high-resolution spatiotemporal data—specifically reflectivity and radial velocity—that captures the internal structure and motion of these systems. Radar echo extrapolation, which predicts future radar reflectivity maps based on historical observations, serves as the fundamental technique for 0–2 h nowcasting [3]. However, the task involves modeling complex physical processes from high-dimensional, noisy, and non-stationary data streams, which remains a formidable challenge at the intersection of atmospheric science and signal processing [4].

Mathematically, radar echo extrapolation is formulated as a spatiotemporal sequence forecasting problem. Traditional methods, such as Optical Flow [5], rely on the assumption of brightness constancy and smooth motion fields. While effective for rigid body motion, these assumptions often fail to capture the fluid nature of atmospheric phenomena, where echo intensity fluctuates due to convection initiation, growth, and dissipation. Numerical Weather Prediction (NWP) models, based on fluid dynamics equations, offer physical consistency but are often computationally too expensive and slow for the rapid update cycles required in nowcasting [6]. Consequently, data-driven deep learning approaches have emerged as a promising alternative, capable of learning complex non-linear mappings directly from historical radar sequences.

Despite significant progress, deep learning models still exhibit distinct scientific limitations when applied to radar sensor data, particularly concerning the fidelity of extrapolated echoes in high-intensity regions. Specifically, deep learning-based extrapolation often struggles to simultaneously mitigate intensity attenuation in core convective regions (echoes > 35 dBZ) and maintain spatiotemporal consistency over longer forecast horizons (e.g., beyond 1 h). General models tend to underestimate sparse but high-value severe weather signals while suffering from error accumulation, which leads to the dissipation or unrealistic deformation of weather systems as prediction time steps increase. A primary reason for these shortcomings is that many existing architectures fail to adequately decouple the complex interactions between spatial morphology and temporal evolution. When these entangled features propagate through deep networks, high-frequency details representing severe convection are often lost or degraded.

To alleviate these inherent limitations and improve the application of deep learning models for radar sensor data, this paper proposes a novel Axial Fusion and Tri-Axis Factorized Attention Network (AFTA-Net). The model aims to improve the predictive accuracy of severe convective weather by employing refined feature recalibration mechanisms. The main contributions of this paper are summarized as follows:

1. We propose an Axial Fusion Block (AFB), which adopts a “divide-and-conquer” strategy using parallel spatiotemporal convolutional structures to decouple sensor features. This effectively separates temporal evolution from spatial structural characteristics, avoiding the computational redundancy of traditional 3D convolutions while preserving structural locality.
2. We design a Tri-Axis Factorized Attention (TAFA) module. Unlike pseudo-“3D attention” mechanisms that imply volumetric modeling without elevation data, TAFA innovatively performs feature recalibration by sequentially decomposing the data tensor along three dimensions: Time (T), Channel (C), and Spatial (S). This mechanism effectively decouples the feature representation space to simulate the evolution process of atmospheric systems and precisely localizes high-value severe convective features.
3. Extensive experiments on the Jiangsu radar dataset demonstrate that AFTA-Net outperforms representative baselines in predicting severe convective intensity and maintaining spatiotemporal consistency, verifying its potential as a robust sensor signal processing tool.

## 2. Related Works

Radar echo extrapolation aims to predict future reflectivity images from historical observation sequences, serving as a core technique for zero-to-two-hour short-term nowcasting. The essence of this task is modeling high-dimensional, non-stationary streams of radar sensor data to capture the complex nonlinear atmospheric dynamics within. This section systematically reviews related research progress, categorized into traditional methods and deep learning-based methods.

### 2.1. Traditional Methods

Traditional radar echo extrapolation primarily relies on physical assumptions and signal processing techniques, without involving learnable deep neural networks.

One representative approach is the Optical Flow method, which performs extrapolation by computing motion vector fields between consecutive radar images [5]. While computationally efficient and effective for stratiform precipitation with steady motion, it relies on assumptions of brightness constancy (i.e., unchanged echo intensity) and smooth motion. These assumptions are inconsistent with the physical reality of severe convective weather, which involves rapid initiation, intensification, and dissipation processes, leading to significant errors in non-linear scenarios.

Another technical route is Numerical Weather Prediction (NWP) models. Based on fluid dynamics and thermodynamic equations, NWP can provide physically consistent forecasts. However, for 0–2 h nowcasting, NWP has inherent bottlenecks: firstly, its high computational cost makes it difficult to meet the minute-level rapid update cycle required for nowcasting; secondly, uncertainties in initial conditions and the parameterization schemes for microphysical processes limit their ability to capture small- and mesoscale sudden severe convection [6].

### 2.2. Deep Learning-Based Methods

With the rapid development of artificial intelligence, data-driven deep learning models have become the dominant paradigm for radar echo extrapolation. These methods learn spatiotemporal patterns directly from massive historical data, treating nowcasting as a sequence-to-sequence prediction problem.

#### 2.2.1. Recurrent Neural Networks (RNNs)

Early deep learning efforts focused on integrating spatial convolutions into recurrent architectures. The pioneering ConvLSTM [7] achieved end-to-end radar echo prediction by capturing local spatial correlations within LSTM units. Subsequently, models like PredRNN [8] and MIM [9] introduced spatiotemporal memory flow to capture higher-order non-stationarity. However, RNN-based models process data sequentially, which limits parallelization. Moreover, due to the vanishing gradient problem, they often struggle to preserve high-frequency information in convective cores over long forecast horizons, leading to the “blurring” effect.

#### 2.2.2. Convolutional Neural Networks (CNNs)

To improve computational efficiency, pure convolutional architectures such as SimVP [10] were proposed. These models excel in parallel computation and local feature extraction. Beyond architectural design, recent research also focuses on optimizing the training dynamics of such networks. For example, the E-HEOA framework employs an evolutionary hybrid optimization algorithm to automatically tune critical hyperparameters for temporal convolutional networks, thereby enhancing their convergence efficiency and final prediction performance [11]. However, their reliance on local kernels limits their receptive fields. In convective weather prediction, where large-scale systemic motion and local intensification coexist, CNN-based models often fail to capture global motion trends without stacking excessively deep layers, which complicates training.

#### 2.2.3. Attention Mechanisms and Transformers

To break the locality constraints, Transformers and attention-based models have been introduced to model long-range dependencies. Specialized architectures like Rainformer [12] utilize global-local transformers to enhance multi-scale feature extraction. Similarly, Earthformer [13] explores spacetime transformers tailored for Earth system forecasting. In particular, recent studies have also explored Axial Attention [14] to solve spatial dependencies along separate axes, significantly reducing computational costs while maintaining high-resolution detail Furthermore, hybrid architectures are emerging that combine the strengths of Transformers with other paradigms. The RaDiT model integrates a differential attention mechanism within a Vision Transformer to better suppress noise in radar images and is coupled with adversarial training to enhance structural consistency, representing a novel application of differential Transformers to this domain [15]. These models are more attuned to the non-linear dynamics of convective systems compared to traditional CNNs.

#### 2.2.4. Generative and Physics-Informed Models

A significant milestone in recent years is the integration of physical consistency with generative capabilities. NowcastNet [16] combines physical evolution laws with generative networks to produce sharp, realistic convective structures even for extreme precipitation events. Similarly, diffusion-based models like DiffCast [17] utilize residual diffusion to mitigate intensity attenuation.Pushing this frontier further, the latest research begins to leverage full volumetric radar data. The DIFF-3DRformer, for example, pioneers a 3D diffusion model constrained by neural operators encoding the physical equations of advection and continuity, demonstrating superior ability in forecasting the three-dimensional structure and intensity evolution of severe storms compared to previous 2D methods [18]. While these approaches produce visually superior results, they may introduce non-physical artifacts or require significant computational resources. Our AFTA-Net aims to bridge this gap by using axial decoupling to maintain structural integrity without the high inference latency of diffusion models.

## 3. Methodology

### 3.1. Overall Architecture

The AFTA-Net processes input radar sensor data formulated as a 5D tensor of shape B×C×T×H×W, where *B* is the batch size, *C* represents the number of feature channels, *T* is the number of time steps, and H,W are the spatial height and width. For our specific task, the input dimensions are 20×1×128×128, referring to 20 consecutive historical frames (2 h), 1 reflectivity channel, and single-temporal-step data with a grid size of 128×128. The encoder consists of four stages using Axial Fusion Blocks (AFB). The hidden feature dimension *D* (initially 96) is progressively scaled to 2D, 4D, and 8D (i.e., 192, 384, 768) as the spatial resolution is downsampled, allowing the model to capture deep systemic motion trends.

**Figure 1:**
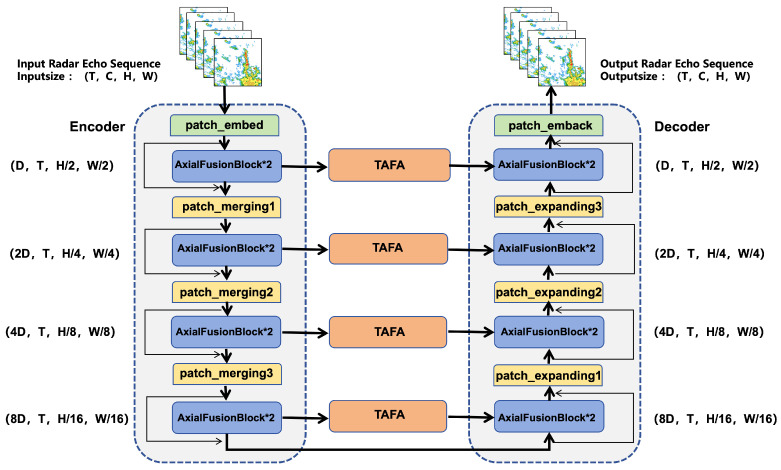
The overall architecture of the proposed AFTA-Net. The network processes a sequence of radar echo frames through a symmetric encoder–decoder structure enhanced by Axial Fusion Blocks (AFB) and Tri-Axis Factorized Attention (TAFA) skip connections. The arrows in the figure indicate data flow, and the asterisks denote multiplicative relationships.

The proposed AFTA-Net adopts a symmetric encoder–decoder architecture explicitly customized for high-dimensional spatiotemporal extrapolation of radar sensor data. As illustrated in Figure 1, the model accepts a discretized spatiotemporal slice tensor of shape 20×1×128×128 as input. Specifically, the encoder consists of four stages of Axial Fusion Blocks (AFB), designed to decouple the high temporal correlations and complex spatial patterns inherent in meteorological sensor streams. The processing flow initiates with a 3D Patch Embedding layer combined with a convolutional layer for initial projection (C=96). In the first three stages, spatial downsampling is performed via strided patch embedding to double the channel depth, while the fourth stage maintains resolution and expands channels to 4C to capture deep systemic motion trends. As a critical bridge connecting the encoder and decoder, the Tri-Axis Factorized Attention (TAFA) module is integrated into the skip connections (see Figure 1). By sequentially recalibrating features along the time, channel, and spatial axes, TAFA effectively enhances the representation of critical convective features. The decoder mirrors the encoder’s topology, utilizing a Patch Expanding mechanism containing linear projection and pixel shuffling to progressively restore spatiotemporal resolution [19]. Residual connections are introduced before each AFB module to enforce residual mapping learning and prevent feature degradation. Finally, a 3D transposed convolution layer reconstructs features back to the original input dimensions, generating the predicted radar echo sequence. It is important to clarify that the “physics-aware” nature of AFTA-Net refers to a conceptual alignment with atmospheric processes through deliberate architectural design, rather than the explicit enforcement of governing physical equations. Specifically, we introduce structural inductive biases that reflect fluid dynamics behavior through a spatiotemporal decoupling strategy. This ensures that the network learns feature representations that maintain physical consistency with the evolution of convective systems.

### 3.2. Axial Fusion Block (AFB)

The AFB achieves explicit spatiotemporal decoupling by processing the tensor along separate axes. 1D temporal convolutions handle the *T* dimension to capture kinematic advection, while 2D spatial convolutions handle the H×W axes for morphological extraction. This design prevents the entanglement of motion and shape, which is a primary cause of blurring in convective core prediction.

**Figure 2:**
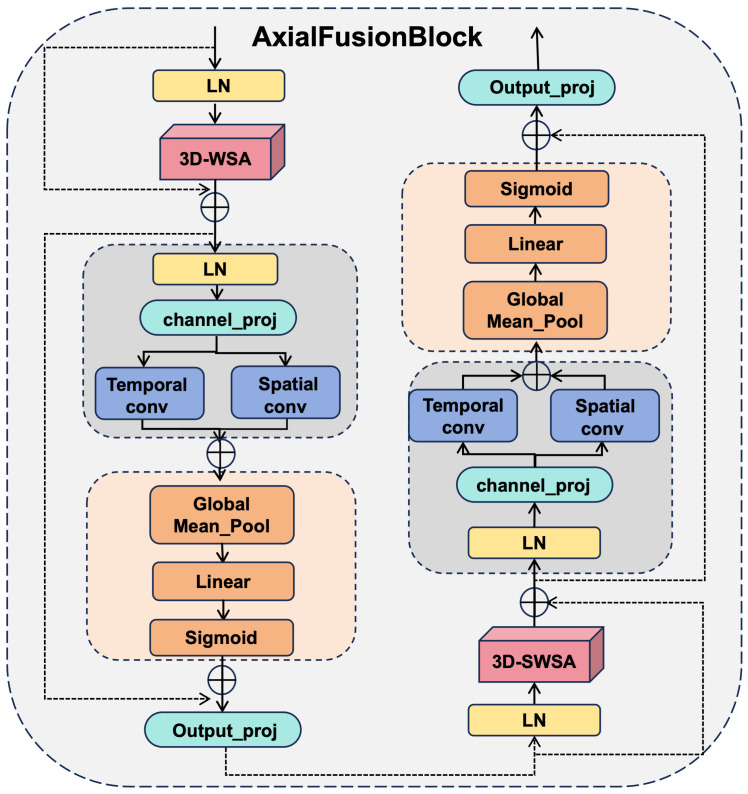
Architecture of the Axial Fusion Block (AFB). The block integrates 3D Window/Shifted-Window Attention with a specialized feed-forward path that employs parallel axial convolutions for spatiotemporal decoupling and a global gating mechanism for feature recalibration. The arrows in the diagram indicate the direction of data flow.

To overcome the inherent limitations of traditional multi-layer perceptron (MLP) modules in transformer models when processing high-dimensional spatio-temporal radar data, this paper proposes the Axial Fusion Block (AFB). Standard MLPs typically flatten inputs into one-dimensional vectors, which destroys structural locality and results in uniform non-linear transformations across all positions, limiting the capability to decouple complex local spatio-temporal patterns.As illustrated in Figure 2, the AFB is designed as a cohesive dual-branch residual architecture that facilitates cross-window information interaction by alternating between 3D Window-based and Shifted-Window-based Multi-Head Self-Attention (3D-WSA/SWSA) [20] in consecutive blocks. Within each module, the standard MLP is replaced with a physics-aware axial processing strategy. The input tensor $X\in R^{B\times C\times T\times H\times W}$ first undergoes parallel axial convolution decomposition: 1D temporal convolutions capture motion trends, while 2D spatial convolutions extract morphological features, thereby achieving explicit spatiotemporal decoupling. Subsequently, a dynamic feature selection mechanism aggregates global context via mean pooling to generate gating values, adaptively recalibrating channel weights to amplify critical meteorological signals. This design reflects the physical hypothesis that atmospheric motion can be conceptually decomposed into temporal advection trends and spatial structural deformations. By employing parallel axial convolutions, the AFB achieves explicit spatiotemporal decoupling, effectively guiding the model to respect the kinematic properties of fluid flow. The overall computation within an AFB unit involves two distinct stages. The first is feature extraction via attention mechanisms:

(1) $$X^{\prime }=3D\text{-}\mathrm{WSA}/\mathrm{SWSA}\left(\mathrm{LN}\left(X\right)\right)+X$$

Subsequently, the integrated feed-forward process is applied:

(2) $$X_{out}={\mathrm{AFB}}_{\mathrm{ffn}}\left(\mathrm{LN}\left(X^{\prime }\right)\right)+X^{\prime }$$

where LN denotes Layer Normalization [21], and ${\mathrm{AFB}}_{\mathrm{ffn}}$ represents the integrated feed-forward process encompassing axial decoupling, convolution, and dynamic gating.

### 3.3. Tri-Axis Factorized Attention (TAFA)

TAFA factorizes the attention computation into three sequential steps: (1) Time Attention (*T*): Identifies key frames of convective initiation; (2) Channel Attention (*C*): Recalibrates the importance of different meteorological feature maps; (3) Spatial Attention (*S*): Localizes the exact grid coordinates of the convective core.

**Figure 3:**
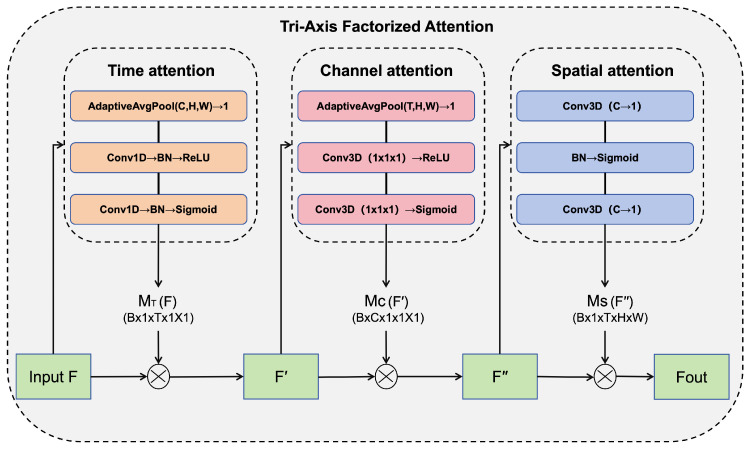
Diagram of the Tri-Axis Factorized Attention (TAFA) module. The input features are sequentially processed through three sub-modules: Time Attention, Channel Attention, and Spatial Attention, progressively refining the feature representation. The arrows in the diagram indicate the direction of data flow.

To reinforce information exchange between the encoder and decoder and mitigate the loss of high-frequency details during upsampling, this paper designs the Tri-Axis Factorized Attention (TAFA) module. As illustrated in Figure 3, TAFA employs a sequential “divide-and-conquer” strategy to factorize complex spatiotemporal dependencies into three recalibration sub-processes along the Time (T), Channel (C), and Spatial (S) axes. Let the input feature map be denoted as $F\in R^{B\times C\times T\times H\times W}$.

Unlike methods that attempt to model physical 3D atmospheric structures without elevation data, our approach focuses on decoupling the feature representation space. The recalibration process begins with the Time Attention branch, which highlights key evolutionary frames. It first compresses the spatial and channel dimensions into a time descriptor via adaptive average pooling (AdaptiveAvgPool(C,H,W)→1), followed by two 1D convolution layers with Batch Normalization (BN) and non-linear activations (ReLU and Sigmoid) to generate the time attention map $M_{T}\left(F\right)\in R^{B\times 1\times T\times 1\times 1}$. This map modulates the input feature *F* to produce the time-refined feature $F^{\prime }$. Subsequently, the Channel Attention branch identifies critical meteorological features by squeezing spatiotemporal dimensions (AdaptiveAvgPool(T,H,W)→1) and exciting channel inter-dependencies using two 1×1×1 3D convolutions (separated by ReLU and ending with Sigmoid), yielding the channel attention map $M_{C}\left(F^{\prime }\right)\in R^{B\times C\times 1\times 1\times 1}$ which refines $F^{\prime }$ into $F^{\prime \prime }$. Finally, the Spatial Attention branch localizes high-intensity echo regions. It compresses the channel dimension to 1 using a 3D convolution, processes the spatial map through Batch Normalization and Sigmoid activation to generate the spatial attention map $M_{S}\left(F^{\prime \prime }\right)\in R^{B\times 1\times T\times H\times W}$, and applies it to $F^{\prime \prime }$ via a final 3D convolution to produce the output $F_{out}$. By factorizing the tensor, the model effectively isolates temporal evolution patterns from spatial appearances.

The sequential arrangement of Time → Channel → Spatial attention is motivated by the physical priority inherent in atmospheric evolution. In radar echo sequences, the temporal dimension (*T*) carries the most critical motion information; by recalibrating the time axis first, the model effectively filters out temporal noise and identifies key evolutionary frames. Channel attention (*C*) follows to refine the physical feature importance of these frames, while Spatial attention (*S*) performs fine-grained localization of convective cores. Compared to an alternative “Spatial-First” strategy, which may be susceptible to background clutter, our “Time-First” approach ensures that the subsequent feature recalibration is anchored on robust dynamic trends, thereby enhancing the stability of long-term extrapolation. The recalibration process is mathematically formulated as a sequential factorization along three axes.

The Time Attention branch modulates temporal dependencies:

(3) $$F^{\prime }=F⊗\sigma \left(\mathrm{BN}\left({\mathrm{Conv}1D}_{2}\left(\delta \left(\mathrm{BN}\left({\mathrm{Conv}1D}_{1}\left({\mathrm{Pool}}_{C,H,W}\left(F\right)\right)\right)\right)\right)\right)\right)$$

The Channel Attention branch refines inter-channel features:

(4) $$F^{\prime \prime }=F^{\prime }⊗\sigma \left({\mathrm{Conv}3D}_{2}\left(\delta \left({\mathrm{Conv}3D}_{1}\left({\mathrm{Pool}}_{T,H,W}\left(F^{\prime }\right)\right)\right)\right)\right)$$

The Spatial Attention branch localizes high-intensity echo regions:

(5) $$F_{out}=F^{\prime \prime }⊗{\mathrm{Conv}3D}_{out}\left(\sigma \left(\mathrm{BN}\left({\mathrm{Conv}3D}_{in}\left(F^{\prime \prime }\right)\right)\right)\right)$$

where ⊗ denotes element-wise multiplication, Pool represents Adaptive Average Pooling along specified dimensions, δ is the ReLU function, and σ is the Sigmoid function.

## 4. Experiments

### 4.1. Dataset

**Figure 4:**
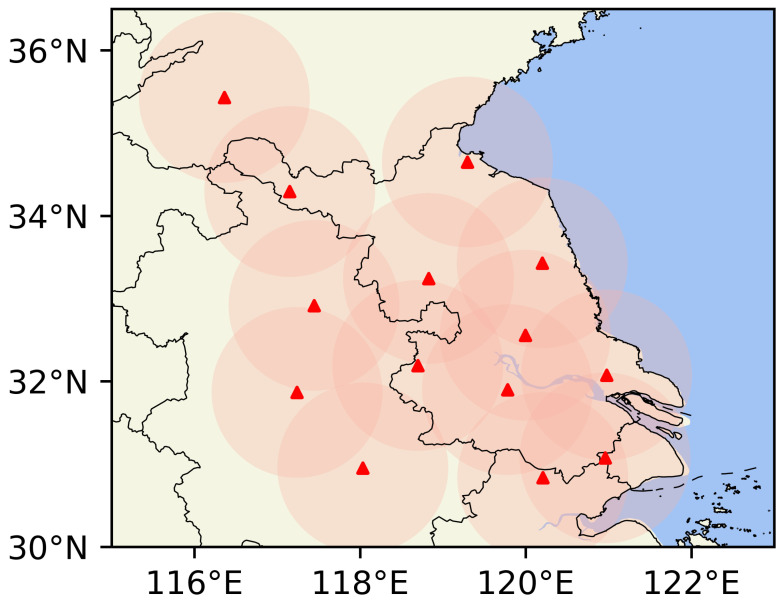
Geographical context of the study area. This map depicts the location of Jiangsu Province, the spatial distribution of S-band radar stations (indicated by red triangles), and the combined coverage boundaries of the dataset.

As shown in Figure 4, this study focuses on the target area covering most of Jiangsu Province in China and its upstream region in Anhui Province. The dataset utilized in this study is derived from the “2022 Jiangsu Meteorological AI Algorithm Challenge.” It is constructed based on observational records from multiple S-band dual-polarization weather radars across Jiangsu Province, collected between April and September 2019. To ensure data reliability, the raw observational data underwent a rigorous quality control process. First, a fuzzy logic-based classifier was employed to effectively eliminate non-meteorological echo interference (e.g., ground clutter, biological scatterers) [22]. Second, a large-scale composite reflectivity product was generated using multi-radar mosaicking technology. Finally, we implemented a secondary screening strategy to further remove clear-air echoes. The processed data exhibits a regular grid structure with a spatial resolution of 0.01° (grid dimensions of 480×560 pixels), a temporal resolution of 6 min, and a radar echo intensity range of 0–70 dBZ.

To tailor the data for the spatiotemporal sequence prediction task, we adopted a sliding window strategy for slice sampling, setting the total window duration to 4 h (covering 40 time steps). Specifically, the first 20 time steps (historical 2 h) serve as the model input, while the subsequent 20 time steps (future 2 h) function as the prediction Ground Truth. Furthermore, to optimize computational efficiency and accommodate model input requirements, all radar echo images were resized to a resolution of 128×128 via bilinear interpolation, and pixel values were normalized to the [0,1] interval.

Following this processing pipeline, the final constructed dataset comprises 12,516 sequences. To ensure a rigorous evaluation, we adopted a random split strategy: 10,765 sequences were allocated for training, 1292 sequences served as the validation set for hyperparameter tuning and early stopping, and the remaining 459 sequences were strictly reserved as a held-out independent test set. Crucially, all quantitative results subsequent analyses are derived exclusively from this independent test set to prevent data leakage and ensure fair comparison.

### 4.2. Implementation Details

To ensure the reproducibility and fairness of experimental results, all models were subjected to a uniform experimental protocol. Specifically, a fixed random seed (1) was employed to initialize all network weights and dataset partitions, while data shuffling strategies were synchronized to guarantee that all baseline models encountered identical sequence batches during each training epoch. All experiments were implemented using the PyTorch (1.12.0) deep learning framework on NVIDIA RTX 4090 GPUs (24 GB). For standardized training, all models were trained for 50 epochs with a batch size of 4, an initial learning rate of $10^{-3}$, and the Adam optimizer [23]. Regarding the optimization objective, we utilized a combined loss function of Mean Squared Error (MSE) and Mean Absolute Error (MAE). This hybrid approach was chosen because exclusive use of MSE may result in vanishingly small gradients that hinder effective training, whereas MAE alone may lead to the loss of essential data feature details [24]. The formula for the loss function is as follows:

(6) $$\begin{array}{cc} \mathrm{MSE} & =\frac{1}{T\times H\times W}\sum_{t=1}^{T}\sum_{h=1}^{H}\sum_{w=1}^{W}{\left(Y_{t,h,w}-{\tilde{Y}}_{t,h,w}\right)}^{2} \end{array}$$

(7) $$\begin{array}{cc} \mathrm{MAE} & =\frac{1}{T\times H\times W}\sum_{t=1}^{T}\sum_{h=1}^{H}\sum_{w=1}^{W}\left|Y_{t,h,w}-{\tilde{Y}}_{t,h,w}\right|\; \end{array}$$

(8) $$\begin{array}{cc} L & =\mathrm{MSE}+\mathrm{MAE} \end{array}$$

where $Y_{t,h,w}$ denotes the actual radar echo value of the target image sequences with pixel coordinates (h,w) at timestamp *t*, and ${\tilde{Y}}_{t,h,w}$ is the corresponding predicted value. *T* is the total length of the predicted sequence, while *H* and *W* are the height and width of the radar image, respectively.

### 4.3. Evaluation Metrics

To quantitatively assess the model’s ability to predict future radar echoes, we employed standard meteorological verification metrics, including the Critical Success Index (CSI) [25], Probability of Detection (POD) [26], False Alarm Ratio (FAR) [27], and Heidke Skill Score (HSS) [28]. Furthermore, to evaluate the visual quality and structural consistency of the predicted images, we utilized image quality metrics such as Peak Signal-to-Noise Ratio (PSNR) and Structural Similarity Index Measure (SSIM) [29].

The evaluation procedure follows standard practice in radar meteorology. The predicted and observed radar echo images are binarized according to specific reflectivity thresholds (τ). Specifically, the metrics (CSI, POD, FAR, HSS) are calculated in a pixel-wise manner. For each pixel in the radar image, the evaluation logic is defined based on a contingency matrix (Table 1): True Positive (TP), False Negative (FN), False Positive (FP), and True Negative (TN).

Based on the contingency matrix, the meteorological metrics are calculated as follows:

(9) $$\mathrm{CSI}=\frac{\mathrm{TP}}{\mathrm{TP}+\mathrm{FN}+\mathrm{FP}}$$

(10) $$\mathrm{POD}=\frac{\mathrm{TP}}{\mathrm{TP}+\mathrm{FN}}$$

(11) $$\mathrm{FAR}=\frac{\mathrm{FP}}{\mathrm{TP}+\mathrm{FP}}$$

(12) $$\mathrm{HSS}=\frac{2\times (\mathrm{TP}\times \mathrm{TN}-\mathrm{FP}\times \mathrm{FN})}{\left(\mathrm{TP}+\mathrm{FN})(\mathrm{FN}+\mathrm{TN})+(\mathrm{TP}+\mathrm{FP})(\mathrm{FP}+\mathrm{TN}\right)}$$

CSI and POD range from 0 to 1, where higher values indicate better performance. FAR measures the fraction of predicted events that did not occur, with lower values being better. HSS ranges from −1 to 1, assessing forecast skill relative to random chance.

Additionally, PSNR and SSIM are defined as:

(13) $$\mathrm{PSNR}=10\cdot \log_{10}\left(\frac{{\mathrm{MAX}}_{I}^{2}}{\mathrm{MSE}}\right)$$

(14) $$\mathrm{SSIM}\left(x,y\right)=\frac{\left(2\mu_{x}\mu_{y}+C_{1}\right)\left(2\sigma_{xy}+C_{2}\right)}{\left(\mu_{x}^{2}+\mu_{y}^{2}+C_{1}\right)\left(\sigma_{x}^{2}+\sigma_{y}^{2}+C_{2}\right)}$$

In Equation (13), ${\mathrm{MAX}}_{I}$ represents the maximum possible pixel value (e.g., 255 for 8-bit images or 1.0 for normalized data), and MSE is the Mean Squared Error between the predicted and ground truth images. In Equation (14), *x* and *y* represent the predicted and ground truth images; $\mu_{x},\mu_{y}$ are local means, $\sigma_{x},\sigma_{y}$ are local standard deviations, and $\sigma_{xy}$ is the cross-covariance. $C_{1}$ and $C_{2}$ are constants to stabilize the division. Higher PSNR and SSIM values indicate better image reconstruction quality and structural similarity.

In this study, thresholds of 10, 20, and 30 dBZ were selected to evaluate meteorological metrics. Our objective is to maximize CSI, POD, PSNR, and SSIM while minimizing FAR.

### 4.4. Comparative Experiments

To rigorously validate the superiority of the proposed AFTA-Net in processing complex meteorological sensor data and performing radar echo extrapolation, this study constructed a comprehensive benchmark suite encompassing four mainstream deep learning paradigms. This suite includes: Recurrent Neural Networks (RNNs), represented by PredRNN [8] and MIM [9]; Convolutional Neural Networks (CNNs), such as SimVP [10]; Transformers, including Rainformer [12] and EarthFarseer [30]; and Generative Models, represented by DiffCast [17]. To mitigate biases arising from varying training strategies, all experiments adhered to a standardized protocol: input and prediction sequence lengths were uniformly set to 20 frames (covering a 0–2 h horizon), and models were trained for 50 epochs under identical optimizer configurations and learning rate schedules to ensure a fair comparison.

Table 2 presents the quantitative evaluation results of different extrapolation methods on the Jiangsu radar dataset. The proposed AFTA-Net achieves superior CSI scores across all intensity thresholds. SimVP exhibits relatively limited performance, which can be attributed to its pure convolutional architecture and lack of specialized temporal feature extraction mechanisms, leading to significant constraints in long-term (0–2 h) forecasting.

Compared to RNN-based models (PredRNN, MIM), Transformer-based approaches generally maintain better structural integrity. Notably, AFTA-Net demonstrates a statistically significant advantage at the 30 dBZ and 20 dBZ thresholds relative to other models. It effectively addresses the issues of prediction blurring and intensity attenuation in strong echo regions—problems that severely degrade the performance of EarthFarseer and PredRNN at these higher thresholds.

Regarding detection capabilities, the generative model DiffCast exhibits competitive performance in POD. However, its effectiveness in comprehensive metrics (CSI and HSS) falls short of AFTA-Net. This suggests that while generative approaches capture motion, they may introduce inconsistent artifacts in complex flow patterns. In contrast, the HSS analysis confirms the superiority of AFTA-Net in forecast clarity, with scores significantly higher than comparative baselines. This indicates that AFTA-Net generates predictions with higher spatial consistency. The results further verify that AFTA-Net maintains a robust advantage across all thresholds, offering a more reliable balance between detection accuracy (POD) and false alarms (FAR) for severe weather monitoring.

### 4.5. Trend Analysis of Evaluation Metrics

#### 4.5.1. Trend Analysis of Critical Success Index (CSI)

**Figure 5:**
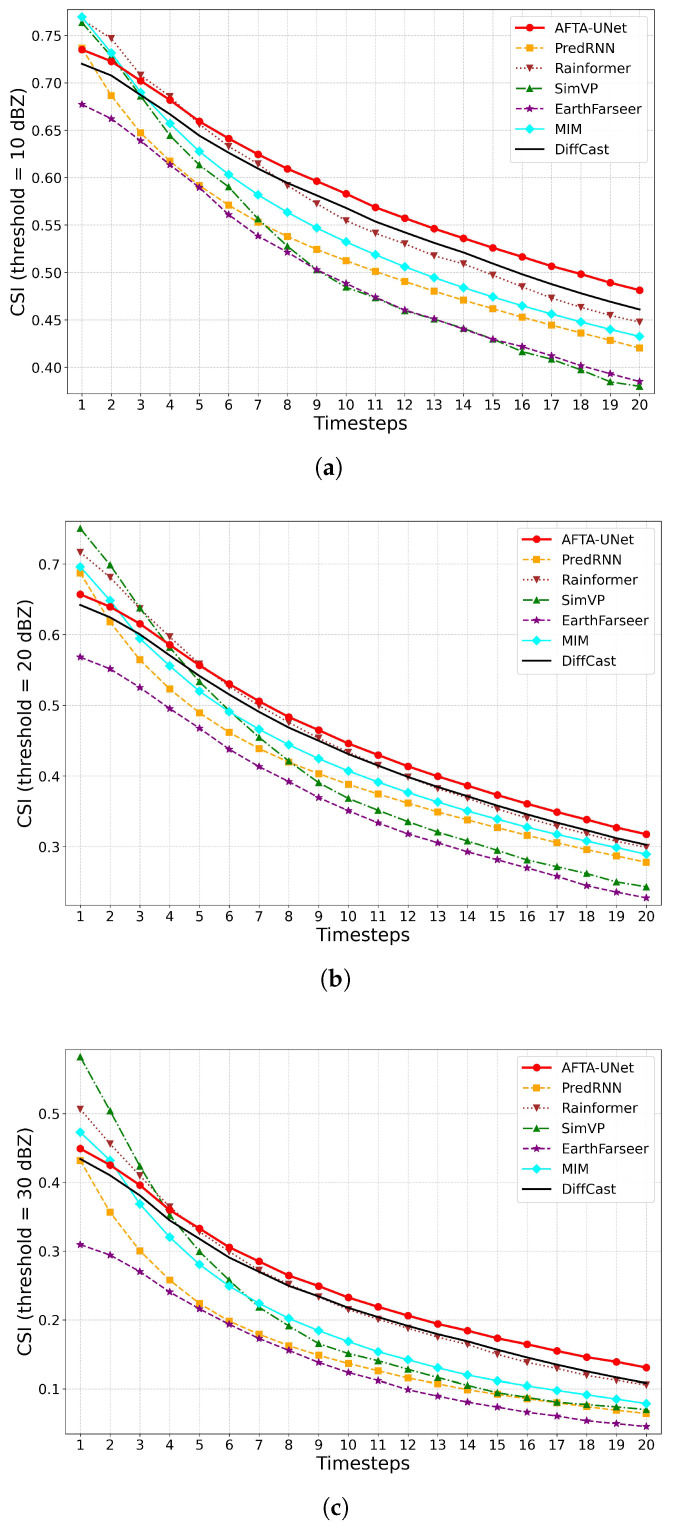
Trend of Critical Success Index (CSI) across varying reflectivity thresholds. The plots illustrate the performance changes over the forecast horizon (0–120 min) at thresholds of 10 dBZ, 20 dBZ, and 30 dBZ on the Jiangsu dataset. (**a**) CSI at 10 dBZ; (**b**) CSI at 20 dBZ; (**c**) CSI at 30 dBZ.

Figure 5 illustrates the trends in the Critical Success Index (CSI) at 10, 20, and 30 dBZ thresholds. All methods exhibit a general decline as the forecast lead time increases. Amidst these fluctuations, AFTA-Net demonstrates exceptional stability; its performance decay curve remains relatively controlled across all 20 time steps. This indicates that the proposed architecture effectively maintains the structural consistency of radar echoes within the 120-min forecast window, achieving results competitive with existing benchmark models.

#### 4.5.2. Trend Analysis of Probability of Detection (POD)

**Figure 6:**
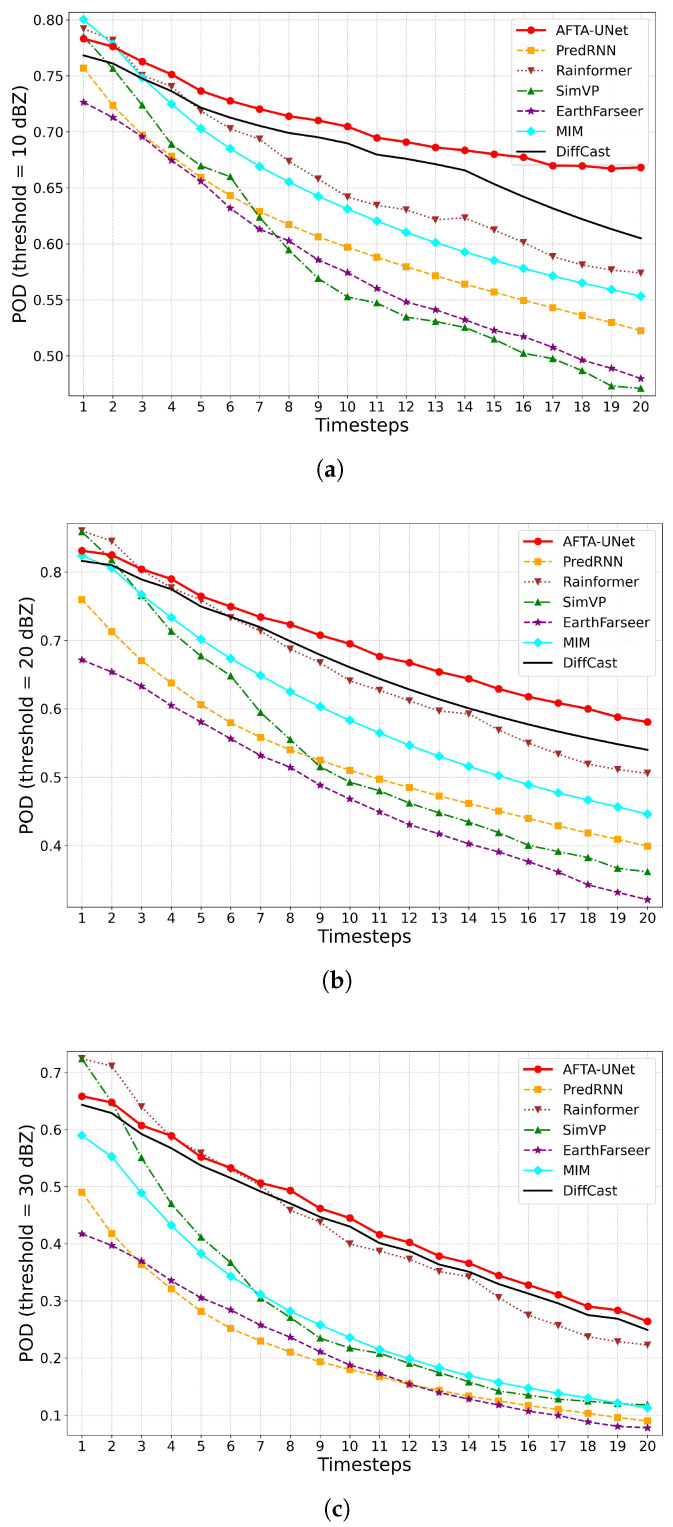
Temporal evolution of Probability of Detection (POD). The trends are displayed for reflectivity thresholds of 10 dBZ, 20 dBZ, and 30 dBZ over the prediction interval. (**a**) POD at 10 dBZ; (**b**) POD at 20 dBZ; (**c**) POD at 30 dBZ.

As illustrated in Figure 6, the probability of detection (POD) naturally diminishes across all methods due to accumulated uncertainties in atmospheric evolution. Against this backdrop, AFTA-Net demonstrates robust feature retention capabilities. Across all tested thresholds, the model consistently maintains POD comparable to or even surpassing leading benchmark methods. This underscores its reliable capability to identify and track valid meteorological signals throughout the entire forecast period.

#### 4.5.3. Trend Analysis of Heidke Skill Score (HSS)

**Figure 7:**
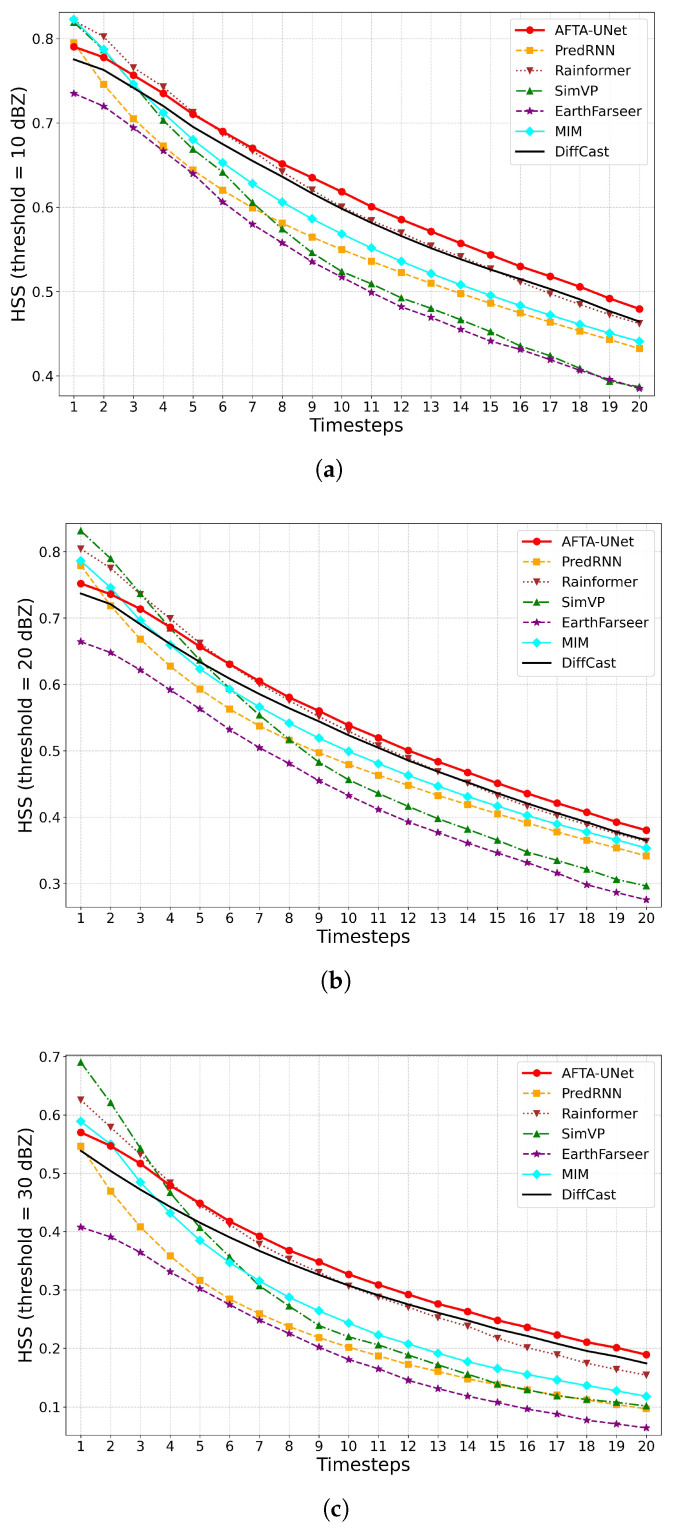
Temporal evolution of Heidke Skill Score (HSS). The plots show the forecasting skill relative to random chance across thresholds of 10 dBZ, 20 dBZ, and 30 dBZ. (**a**) HSS at 10 dBZ; (**b**) HSS at 20 dBZ; (**c**) HSS at 30 dBZ.

Figure 7 presents the Heidke Skill Score (HSS), employed to evaluate the overall forecasting skill relative to random probability. While HSS values generally decline over time, AFTA-Net demonstrates commendable stability. By effectively modelling spatiotemporal dependencies, this architecture maintains a reasonable forecasting level, particularly preserving the structural coherence of radar echoes throughout the forecast run.

#### 4.5.4. Trend Analysis of False Alarm Ratio (FAR)

**Figure 8:**
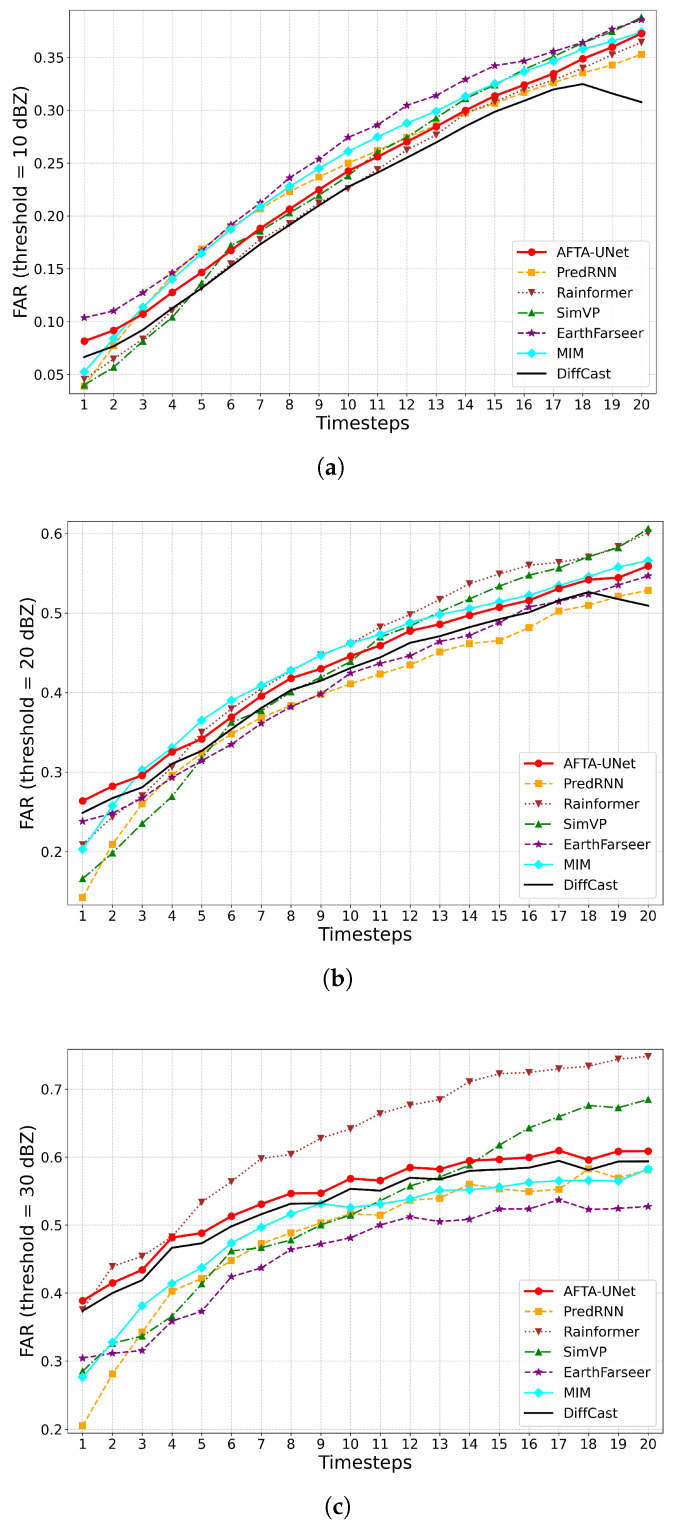
Temporal evolution of False Alarm Ratio (FAR). The changes in false alarm rates are illustrated for thresholds of 10 dBZ, 20 dBZ, and 30 dBZ. (**a**) FAR at 10 dBZ; (**b**) FAR at 20 dBZ; (**c**) FAR at 30 dBZ.

In terms of the False Alarm Rate (FAR) illustrated in Figure 8, all models exhibit an upward trend as the lead time increases. Notably, AFTA-Net does not achieve the absolute lowest FAR in the later stages compared to conservative benchmarks such as DiffCast. This slight increase in FAR stems from a strategic design trade-off: AFTA-Net employs responsive feature recalibration (TAFA) to prevent intensity decay in high-impact echoes. While this mechanism prioritizes the retention of convective cores and enhances sensitivity to potential severe weather events (thereby improving POD), it may inadvertently preserve minor marginal features, resulting in a marginal rise in false alarms.

### 4.6. Evaluation of Image Quality Metrics

**Figure 9:**
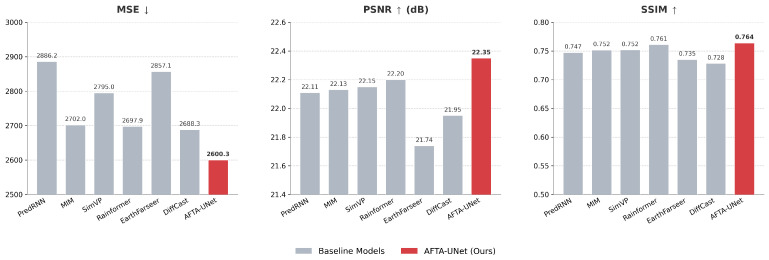
Comparison of image quality metrics on the test set. The bar charts illustrate the average performance of AFTA-Net (Ours, highlighted in red) against six baseline models. (**Left**) Mean Squared Error (MSE). (**Middle**) Peak Signal-to-Noise Ratio (PSNR). (**Right**) Structural Similarity Index (SSIM).

In addition to meteorological skill scores, we utilized Mean Squared Error (MSE), Peak Signal-to-Noise Ratio (PSNR) and Structural Similarity Index (SSIM) to assess the perceptual quality of extrapolated radar echoes. Figure 9 presents the quantitative results of AFTA-Net alongside several baseline models.

In terms of MSE, which reflects pixel-level deviations, AFTA-Net achieves a value of 2600.3. This is lower than the baseline models, which range from 2688.3 to 2886.2, suggesting that our method effectively reduces prediction errors in the generated frames.

Regarding signal fidelity, AFTA-Net attains a PSNR score of 22.35 dB. This performance is comparable to, and slightly higher than, the second-best model, Rainformer (22.20 dB), while showing a distinct improvement over EarthFarseer (21.74 dB). The results indicate that the proposed model maintains a reasonable signal-to-noise ratio during extrapolation.

For structural consistency, the SSIM results show that AFTA-Net reaches a score of 0.764. This is marginally higher than Rainformer (0.761) and shows an advantage over DiffCast (0.728). These metrics imply that the integration of the Axial Fusion Block (AFB) and Tri-Axis Factorized Attention (TAFA) contributes to preserving structural details in the radar echoes. Overall, the quantitative data demonstrates that AFTA-Net achieves competitive performance in terms of image quality metrics.

### 4.7. Analysis of Computational Complexity and Operational Efficiency

Table 3 presents a detailed quantitative comparison of computational complexity between AFTA-Net and several state-of-the-art baseline models. To ensure the rigor and fairness of the evaluation, all experiments were conducted on a unified hardware platform equipped with an NVIDIA RTX 4090 GPU. The testing configuration was standardized as a sequence-to-sequence task (20 input frames and 20 predicted frames) with a spatial resolution of 128×128 pixels, thereby achieving high comparability for Floating Point Operations (FLOPs).

In terms of model capacity and parameter efficiency, AFTA-Net comprises 43.90 M parameters, representing a 22.0% reduction compared to Rainformer (56.27 M), which also utilizes a Transformer architecture. This significant parameter optimization validates the superiority of the proposed Axial Fusion mechanism in capturing high-dimensional spatiotemporal features. By decomposing complex global attention into multi-axial feature fusion, this mechanism more effectively suppresses structural parameter redundancy, thereby substantially enhancing the representational efficiency per parameter.

Despite sharing comparable FLOPs with PredRNN and MIM, AFTA-Net achieves an inference time of just 50.11 ms, representing a 3.3x speedup over MIM (168.71 ms). This significant acceleration is attributed to AFTA-Net’s non-recursive architecture, which eliminates the sequential dependency bottlenecks inherent in Recurrent Neural Networks (RNNs), thereby fully leveraging the GPU’s parallel computing potential. In contrast, the EarthFarseer model incurs higher computational latency, primarily due to its substantial parameter scale (175.53 M) and intensive computational load (550.73 G FLOPs).

Although the lightweight SimVP model (14.41 M) possesses an extremely high inference speed, its purely 2D convolutional structure has inherent modeling limitations in capturing complex 3D evolutionary features. Furthermore, compared with the generative architecture DiffCast based on diffusion models, although the latter reduces inference time through an efficient sampling algorithm (DDIM), its iterative denoising nature results in a total computational cost (335.03 G FLOPs) that far exceeds that of AFTA-Net. In summary, AFTA-Net achieves a superior balance between prediction accuracy and computational cost while maintaining precise short-term forecasting performance.

### 4.8. Spatial Resolution Sensitivity Analysis

To address the impact of spatial discretization on the model’s ability to represent multi-scale meteorological features, we conducted a sensitivity analysis comparing the 128×128 resolution (baseline) with a higher 256×256 resolution. The results are summarized in Table 4 and Table 5.

Evaluation of meteorological skill scores highlights a notable performance disparity at high-intensity thresholds between the two resolutions. For the 30 dBZ threshold, the resolution 128×128 significantly exceeds the 256×256 configuration, achieving a Critical Success Index (CSI) of 0.2506 and a Probability of Detection (POD) of 0.4439, compared to 0.1969 and 0.2765, respectively (Table 4). While the higher resolution yields a marginally lower False Alarm Ratio (FAR) of 0.3796, its ability to accurately detect intense convective cells diminishes sharply. This discrepancy is characteristic of the double penalty effect [31,32] inherent in grid-based verification: although the 256×256 model produces finer spatial features, even minor spatial displacements of localized convective cells lead to disproportionately severe score degradation.

Metrics for image quality and computational efficiency (Table 5) further underscore the trade-off between pixel-level reconstruction and physical forecasting. The 256×256 resolution exhibits superior PSNR (22.89 dB) and SSIM (0.813), suggesting higher structural similarity to the ground truth. However, this gain in visual fidelity does not produce a corresponding improvement in meteorological accuracy. Conversely, the significantly lower MSE (2600.3) at the 128×128 scale indicates that the model more effectively preserves the overall intensity distribution of the precipitation field, which is vital for reliable quantitative forecasting.

When considering deployment in real-time environments, the 128×128 configuration offers a decisive advantage in efficiency. Increasing the resolution to 256×256 results in a nearly fourfold surge in both inference latency (from 50.11 ms to 198.76 ms) and computational complexity (from 168.46 G to 673.84 G FLOPs). Given that higher resolution compromises the prediction of the convective core while imposing a substantial computational burden, the resolution of 128 × 128 achieves a better balancing effect.

### 4.9. Qualitative Analysis of Typical Case Study

**Figure 10:**
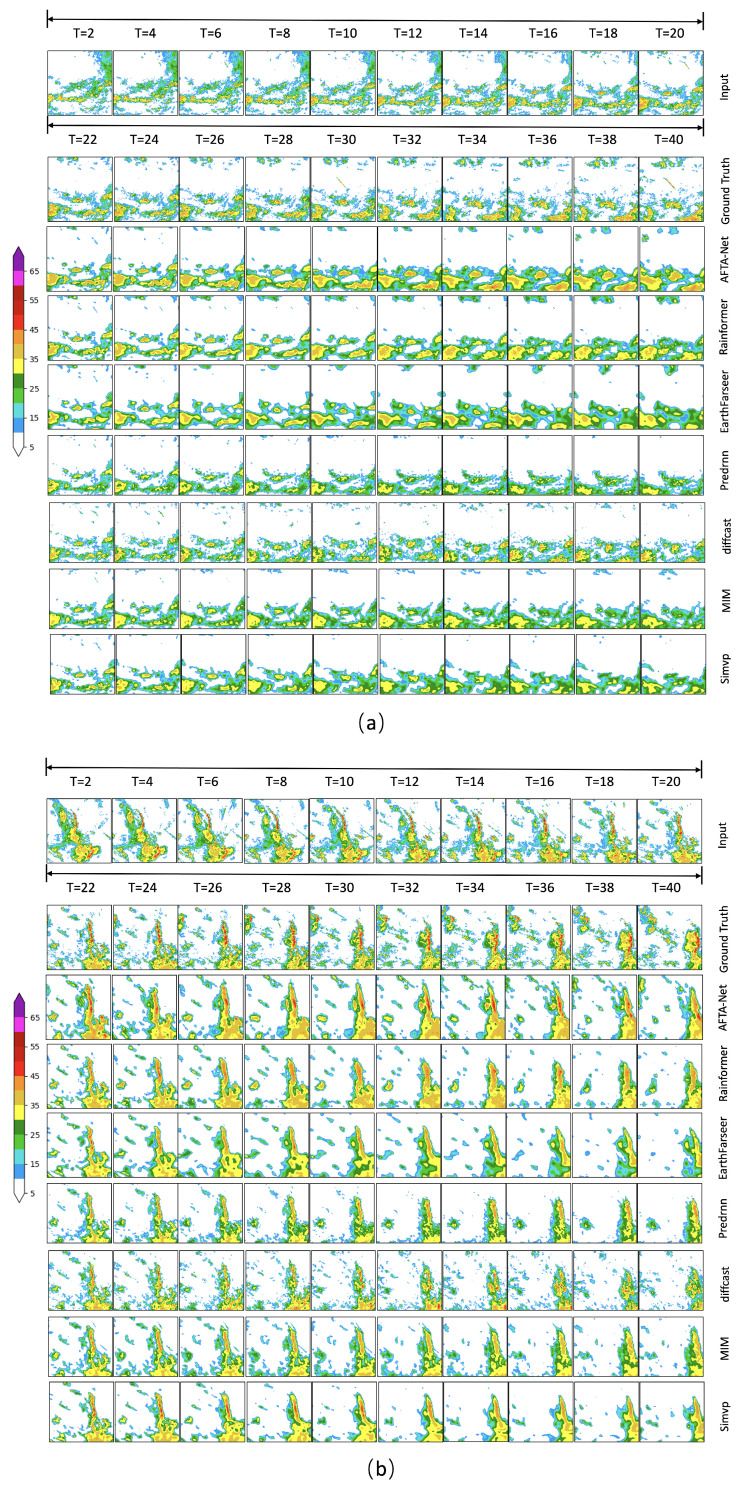
Qualitative comparison of radar echo extrapolation results on two typical severe weather scenarios. (**a**) A convective generation and dissipation process involving scattered cells. (**b**) A linear convective system (squall line) with high-intensity echoes. The rows display the Ground Truth and predictions from seven comparative models.

Figure 10 presents qualitative assessments demonstrating the predictive performance of various models under different weather conditions: (a) isolated convective cells incorporating rapid formation and dissipation processes and (b) squall line systems exhibiting pronounced linear characteristics.

In Case (a), the radar echoes exhibit the dynamic rapid generation and dissipation of dispersed convective cells. Compared to the Ground Truth, baseline models show limitations in capturing these rapidly evolving high-intensity echoes. The CNN-based SimVP, limited by its temporal feature extraction, exhibits obvious blurring from T=22 and degenerates into a smooth background after T=30. RNN-based models suffer from significant “intensity decay”: MIM shows a sharp intensity drop at T=28, while PredRNN loses significant intensity after T=30, missing almost all signals >35 dBZ by T=40. Transformer-based models maintain contours well before T=30, but Rainformer blurs edge echoes after T=32, and EarthFarseer shrinks the central strong echo range after T=34. Although the generative model DiffCast maintains contrast, it introduces spatial noise starting from T=24, leading to a non-physical distribution in the T=34∼40 phase. In contrast, AFTA-Net effectively captures spatiotemporal evolution throughout the timeline (especially T=36∼40), accurately predicting intensification and dissipation consistent with the Ground Truth.

Case (b) demonstrates a linear convective system (squall line) characterized by continuous band-shaped high reflectivity (>35 dBZ). While the Ground Truth shows stable intensity, SimVP barely outlines the profile at T=22 before fracturing at T=24 and completely diffusing after T=30. Among RNN-based models, MIM shows a distinct fracture at T=28, and PredRNN, while maintaining connectivity initially, loses the core strong convection area after T=30 with severe detail loss. Transformer-based models remain identifiable until T=32, but subsequently, Rainformer shows abnormal tail dissipation after T=36, and EarthFarseer appears loose after T=38. DiffCast exhibits severe “dot-like” artifacts after T=32, destroying structural continuity. Notably, AFTA-Net mitigates the “echo intensity decay” problem, maintaining perfect linear continuity and accurate high-intensity gradients from T=22 to T=40, yielding a morphology closest to real observations.

### 4.10. Ablation Study

To empirically verify the effectiveness of the proposed modules within AFTA-Net, we conducted ablation studies based on the results shown in Table 6. We established two variants: W/O TAFA (without the Tri-Axis Factorized Attention module) and W/O AFB (replacing the Axial Fusion Block with standard convolutional layers).

Table 6 summarizes the 120-min extrapolation performance evaluated on the Jiangsu dataset. The complete AFTA-Net configuration demonstrates scoring metrics superior to the ablated architectures across all intensity thresholds. This confirms that the decoupled modeling of temporal evolution and spatial features effectively mitigates mutual interference during the feature learning process. It is worth noting that compared to the W/O TAFA variant, the full AFTA-Net achieves a significantly lower FAR while maintaining a robust POD at the 30 dBZ threshold. This validates the dual advantages of the proposed attention mechanism: enhancing prediction clarity by improving spatial consistency, and elevating the nowcasting precision of severe convective systems, as evidenced by its optimal CSI and HSS scores across all thresholds.

**Figure 11:**
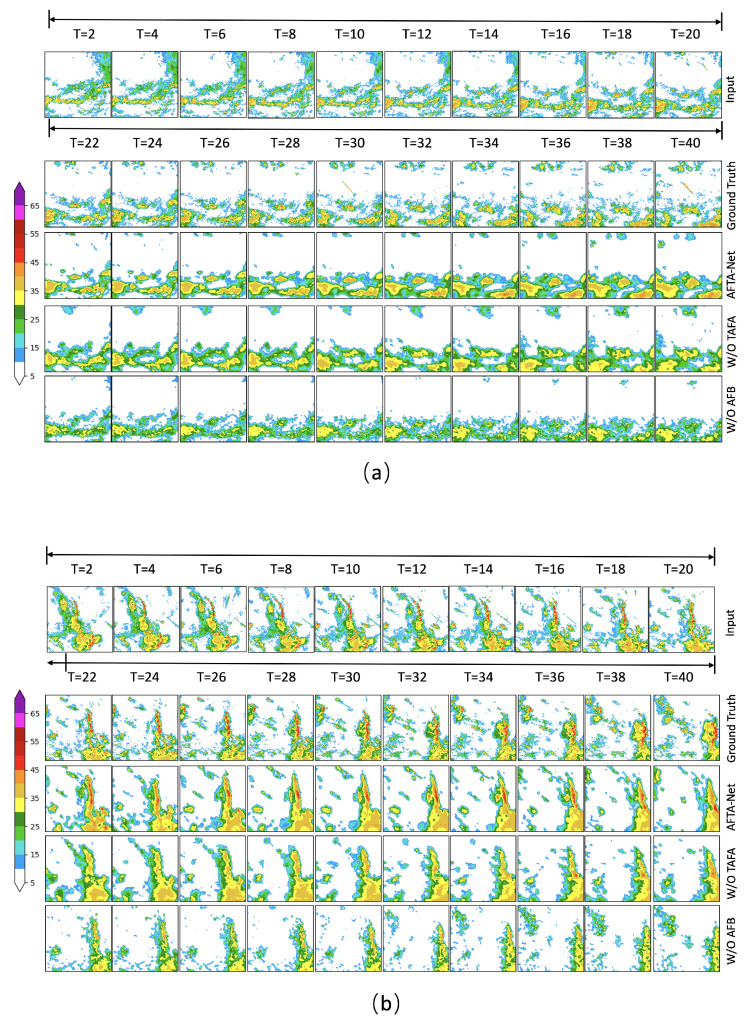
Visual comparison of ablation study results. The figure displays prediction sequences from T=2 to T=40. (**a**) A convective generation and dissipation case. (**b**) A linear convection case. Comparison is made between the full AFTA-Net, W/O TAFA, and W/O AFB variants. AFTA-Net demonstrates superior structural retention and intensity accuracy in both scenarios.

Figure 11 visualizes the impact of the proposed modules across two distinct meteorological scenarios: convective generation/dissipation (Figure 11a) and linear convection (Figure 11b).

In the generation/dissipation case (a), the W/O AFB variant suffers from rapid performance degradation. The root cause lies in the inability of standard convolutional layers to effectively decouple the complex spatiotemporal dependencies of evolving cells, leading to a failure in predicting the initiation and decay of high-intensity cores. Conversely, in the linear convection case (b), while the W/O TAFA variant captures general motion trends, its performance is constrained by insufficient feature recalibration. This results in diffused signals where the high-reflectivity band lacks the compactness and sharp gradients of the Ground Truth. Crucially, by integrating TAFA and AFB, AFTA-Net consistently outperforms both baselines, preserving the structural integrity of the squall line and the dynamic evolution of convective cells. This validates the effectiveness of our dual-module design in refining the structural representation of complex sensor data and ensuring robust long-term extrapolation.

## 5. Conclusions

This study introduces AFTA-Net, a model designed to tackle intensity attenuation and structural inconsistency in radar echo extrapolation. Its superior performance at the 30 dBZ threshold stems from aligning the network architecture with atmospheric fluid physics rather than relying on increased parametric complexity. Unlike conventional deep learning methods where high-frequency details degrade due to entangled spatio-temporal features, AFTA-Net preserves structural integrity through its Axial Fusion Block (AFB), which explicitly decouples temporal dynamics from spatial morphology. This is complemented by the Tri-Axis Factorized Attention (TAFA) mechanism, which acts as a precise recalibration tool to enhance sensitivity to high-intensity convective signals.

From an operational perspective, AFTA-Net offers tangible benefits for nowcasting centers. By balancing predictive accuracy with computational efficiency, the model meets the strict latency requirements of real-time forecasting, ensuring results are generated well before the next radar scan. Specifically, its reliability at the 30 dBZ threshold provides forecasters with a practical objective reference for identifying severe weather, thereby supporting more informed decision-making for disaster mitigation and public safety.

Consequently, future research will focus on integrating multi-source sensor fusion to capture the full stochastic nature of atmospheric dynamics. Specifically, we acknowledge that relying solely on reflectivity limits the model’s ability to distinguish hydrometeor types and correct for attenuation. In future iterations, we aim to incorporate dual-polarization variables, such as Differential Reflectivity (ZDR) and Specific Differential Phase (KDP). ZDR is critical for identifying the phase state and size sorting of hydrometeors (e.g., distinguishing between heavy rain and hail), while KDP offers robust rain rate estimation immune to attenuation and partial beam blocking [4,33]. Incorporating these thermodynamic proxies will allow the network to learn the microphysical lifecycle of convective cells—rather than just their kinematic motion—thereby improving the prediction of storm initiation and decay [34].

## Figures and Tables

**Table 1:** Contingency table for model evaluation metrics.

Prediction	Observation	
	Yes (≥τ)	No (<τ)
Yes (≥τ)	TP	FP
No (<τ)	FN	TN

**Table 2:** Quantitative comparison of AFTA-Net with representative deep learning baselines. The table reports CSI, POD, FAR, and HSS at thresholds of 10, 20, and 30 dBZ, along with their average (Avg). Best results for each metric are highlighted in **bold**. ↑ indicates higher is better, while ↓ indicates lower is better.

Algorithm	CSI ↑				POD ↑				FAR ↓				HSS ↑			
	10	20	30	Avg	10	20	30	Avg	10	20	30	Avg	10	20	30	Avg
EarthFarseer	0.5031	0.3668	0.1422	0.3374	0.5833	0.4763	0.2084	0.4227	0.2613	0.4097	**0.4563**	0.3758	0.5318	0.4445	0.1995	0.3919
PredRNN	0.5282	0.4112	0.1654	0.3683	0.6074	0.5280	0.2089	0.4481	0.2372	**0.3959**	0.4810	**0.3714**	0.5648	0.4988	0.2333	0.4323
DiffCast	0.5750	0.4480	0.2350	0.4193	0.6848	0.6647	0.4300	0.5932	**0.2179**	0.4169	0.5279	0.3876	0.6050	0.5289	0.3199	0.4846
MIM	0.5511	0.4303	0.2009	0.3941	0.6437	0.5980	0.2724	0.5047	0.2481	0.4399	0.4974	0.3951	0.5855	0.5178	0.2771	0.4601
SimVP	0.5169	0.4123	0.2060	0.3784	0.5853	0.5392	0.2849	0.4698	0.2357	0.4276	0.5177	0.3937	0.5532	0.4943	0.2776	0.4417
Rainformer	0.5724	0.4545	0.2410	0.4226	0.6598	0.6552	0.4265	0.5805	0.2246	0.4480	0.6229	0.4318	0.6134	0.5429	0.3296	0.4953
**AFTA-Net**	**0.5891**	**0.4589**	**0.2506**	**0.4329**	**0.7087**	**0.6945**	**0.4439**	**0.6157**	0.2373	0.4343	0.5430	0.4049	**0.6209**	**0.5459**	**0.3430**	**0.5033**

**Table 3:** Quantitative comparison of model complexity. All metrics were measured with a batch size of 4 and an input resolution of 128×128 pixels on an NVIDIA RTX 4090 GPU. ↑ indicates better, ↓ indicates worse, Best results for each metric are highlighted in bold.

Model	Params (M) ↓	FLOPs (G) ↓	Inf. Time (ms) ↓
EarthFarseer	175.53	550.73	202.20
PredRNN	13.92	117.70	93.31
DiffCast	**13.16**	335.03	37.82
MIM	25.65	195.88	168.71
SimVP	14.41	**71.22**	**28.32**
Rainformer	56.27	148.93	33.86
AFTA-Net	43.90	168.46	50.11

**Table 4:** Comparison of meteorological forecast skill metrics between 128×128 and 256×256 spatial resolutions. Metrics are averaged over the 2-h forecast horizon. ↑ indicates better, ↓ indicates worse, Best results for each metric are highlighted in bold.

Threshold	Resolution	CSI ↑	POD ↑	FAR ↓	HSS ↑
10 dBZ	128×128	0.5891	0.7087	0.2373	0.6209
	256×256	0.5560	0.6078	0.1555	0.6021
20 dBZ	128×128	0.4589	0.6945	0.4343	0.5459
	256×256	0.4413	0.5452	0.2887	0.5319
30 dBZ	128×128	**0.2506**	**0.4439**	0.5430	**0.3430**
	256×256	0.1969	0.2765	**0.3796**	0.2731

**Table 5:** Comparison of image quality metrics and computational efficiency between different spatial resolutions. ↑ indicates better, ↓ indicates worse, Best results for each metric are highlighted in bold.

Metric	128×128	256×256
MSE ↓	**2600.3**	8827.2
PSNR ↑	22.35	**22.89**
SSIM ↑	0.764	**0.813**
Inf. Time (ms) ↓	**50.11**	198.76
Params (M) ↓	43.90	43.90
FLOPs (G) ↓	**168.46**	673.84

**Table 6:** Quantitative comparison of ablation variants on the Jiangsu dataset. Metrics (CSI, POD, FAR, HSS) are reported at 10, 20, and 30 dBZ thresholds, along with their average (Avg). Best results for each metric are highlighted in bold. ↑ indicates better, ↓ indicates worse.

Model	CSI ↑				POD ↑				FAR ↓				HSS ↑			
	10	20	30	Avg	10	20	30	Avg	10	20	30	Avg	10	20	30	Avg
**AFTA-Net**	**0.5891**	**0.4589**	**0.2506**	**0.4329**	**0.7087**	**0.6945**	0.4439	**0.6157**	**0.2373**	**0.4343**	**0.5430**	**0.4049**	**0.6209**	**0.5459**	**0.3430**	**0.5033**
W/O TAFA	0.5653	0.4296	0.2188	0.4046	0.6781	0.6656	**0.4585**	0.6007	0.2514	0.4582	0.6125	0.4407	0.6020	0.5172	0.3064	0.4752
W/O AFB	0.5139	0.3519	0.1655	0.3438	0.6520	0.5863	0.3062	0.5148	0.2895	0.5104	0.6847	0.4949	0.5584	0.4672	0.2883	0.4380

## Data Availability

Data is contained within the article or available on request from the corresponding author.
